# Mitochondrial DNA Variants at Low-Level Heteroplasmy and Decreased Copy Numbers in Chronic Kidney Disease (CKD) Tissues with Kidney Cancer

**DOI:** 10.3390/ijms242417212

**Published:** 2023-12-07

**Authors:** Yuki Kanazashi, Kazuhiro Maejima, Todd A. Johnson, Shota Sasagawa, Ryosuke Jikuya, Hisashi Hasumi, Naomichi Matsumoto, Shigekatsu Maekawa, Wataru Obara, Hidewaki Nakagawa

**Affiliations:** 1 Laboratory for Cancer Genomics, RIKEN Center for Integrative Medical Sciences, Yokohama 230-0045, Japan; t236020b@yokohama-cu.ac.jp (Y.K.); kazuhiro.maejima@riken.jp (K.M.); todd.johnson@riken.jp (T.A.J.); shota.sasagawa@riken.jp (S.S.); 2 Department of Human Genetics, Yokohama City University, Yokohama 236-0004, Japan; naomat@yokohama-cu.ac.jp; 3 Department of Urology, Yokohama City University, Yokohama 236-0004, Japan; jikuya@yokohama-cu.ac.jp (R.J.); hasumi@yokohama-cu.ac.jp (H.H.); 4 Department of Urology, Iwate Medical University, Iwate 028-3694, Japan; wadiwadi@dream.com (S.M.); watao@iwate-med.ac.jp (W.O.)

**Keywords:** mitochondrial genome (mtDNA), mitochondrial copy number (mtDNA-CN), chronic kidney disease (CKD), heteroplasmy

## Abstract

The human mitochondrial genome (mtDNA) is a circular DNA molecule with a length of 16.6 kb, which contains a total of 37 genes. Somatic mtDNA mutations accumulate with age and environmental exposure, and some types of mtDNA variants may play a role in carcinogenesis. Recent studies observed mtDNA variants not only in kidney tumors but also in adjacent kidney tissues, and mtDNA dysfunction results in kidney injury, including chronic kidney disease (CKD). To investigate whether a relationship exists between heteroplasmic mtDNA variants and kidney function, we performed ultra-deep sequencing (30,000×) based on long-range PCR of DNA from 77 non-tumor kidney tissues of kidney cancer patients with CKD (stages G1 to G5). In total, this analysis detected 697 single-nucleotide variants (SNVs) and 504 indels as heteroplasmic (0.5% ≤ variant allele frequency (VAF) < 95%), and the total number of detected SNVs/indels did not differ between CKD stages. However, the number of deleterious low-level heteroplasmic variants (pathogenic missense, nonsense, frameshift and tRNA) significantly increased with CKD progression (*p* < 0.01). In addition, mtDNA copy numbers (mtDNA-CNs) decreased with CKD progression (*p* < 0.001). This study demonstrates that mtDNA damage, which affects mitochondrial genes, may be involved in reductions in mitochondrial mass and associated with CKD progression and kidney dysfunction.

## 1. Introduction

Mitochondria are essential cellular organelles in eukaryotes, and the human mitochondrial genome (mtDNA) is a double-stranded circular DNA molecule with a length of 16,569 bp, encoding 13 proteins necessary for oxidative phosphorylation and 22 tRNAs [1,2]. Each mitochondrion possesses multiple mtDNA copies, and any single cell can contain numerous mitochondria. Pathogenic mtDNA variants are commonly heteroplasmic [3] and cause not only mitochondrial diseases [4] but also other diseases such as Alzheimer’s disease, diabetes and cancer [5,6,7,8]. In addition, mitochondrial dysfunction is involved in the pathogenicity of highly prevalent diseases, including neurodegeneration, cardiomyopathies, metabolic disease, obesity and kidney diseases [9,10], because mitochondria play roles in important tasks, such as ATP production, cellular differentiation, pro-inflammatory signaling and apoptosis [10,11]. Therefore, mitochondria represent an important drug target for these common and rare diseases [12].

Kidneys possess one of the highest resting metabolic rates among human organs and require abundant mitochondria to produce sufficient energy to fulfill their various renal functions, such as removing waste products from blood and regulating fluid and electrolyte balance [13,14]. Thereby, mtDNA damage that arises in the context of kidney dysfunction may further exacerbate renal disease and lead to systemic consequences [10,15]. Chronic kidney disease (CKD) is defined as abnormalities of the kidney’s structure or function, presenting for more than three months, which can have broad implications for human health and is now a major global public health problem [16,17]. Several factors contribute to CKD progression, including parenchymal cell loss, chronic inflammation, fibrosis and reduced regenerative capacity of the kidney [18,19]. Recent studies have shown that mtDNA variants can be detected in non-tumor kidney tissue samples as well as kidney tumors in patients with end-stage renal disease (ESRD) [20]. In addition, mitochondrial DNA haplogroups and polymorphisms are associated with CKD, but previous studies focused primarily on homoplasmic variants [21,22,23,24,25]. Therefore, the spectrum of mtDNA variants and mitochondrial heteroplasmy in CKD remains open for further exploration.

In this study, we developed a customized method for mtDNA-specific ultra-deep sequencing (mtDNA-seq) based on long-range PCR (LRPCR) to detect low-level heteroplasmic variants. To identify mtDNA damage related to CKD progression, we performed mtDNA-seq on 77 kidney tissue samples from patients with CKD (stages G1 to G5) and annotated SNVs and indels to assess the accumulation of mtDNA variants and their deleteriousness. We also examined the mtDNA copy number (mtDNA-CN) using quantitative PCR (qPCR) to explore mitochondrial capacity in kidney tissue with CKD.

## 2. Results

### 2.1. mtDNA Sequencing

**Figure 1:**
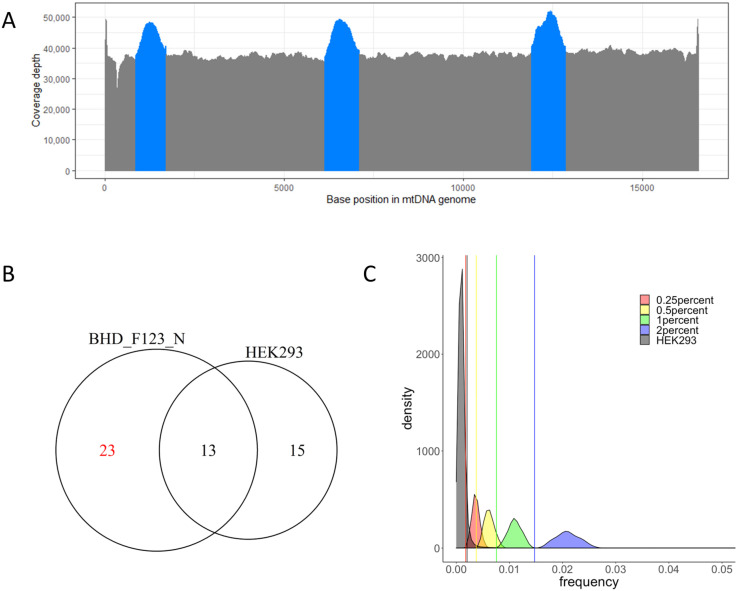
(**A**) Distribution of coverage depth at all mtDNA base positions after enrichment using three pairs of primers. The average was calculated from all sequencing data. Overlapping regions are highlighted in blue. (**B**) Venn diagram shows shared/private homoplasmic SNVs between HEK293 cell line and normal kidney BHD-F123-N. Red color means the private homoplasmic variants in BHD-F123-N used to simulate artificial heteroplasmy via data admixture. (**C**) Frequency of non-reference variants at all positions from HEK293 and 23 artificial heteroplasmic positions from in silico admixture. Each color represents the mixture ratio of admixture or HEK293. Black vertical line indicates the upper limit from HEK293, and the other colored vertical lines indicate the lower confidence limits of 2% (blue), 1% (green), 0.5% (yellow) and 0.25% (red) mixture ratio, respectively.

We obtained a total of 77 frozen kidney tissues from Japanese patients with kidney cancer and CKD (G1: *n* = 16, G2: *n* = 15, G4: *n* = 4, G5: *n* = 42) and performed PCR-based enrichment using three pairs of primers which were designed to produce PCR amplicons of mtDNA [26]. The mtDNA libraries were constructed by mixing the PCR products and sequenced with HiSeq2500 (Illumina, San Diego, CA, USA). Fastq files were pre-processed and aligned with the revised Cambridge Reference Sequence (rCRS, NC_012920.1), which is a general human mitochondrial reference sequence as well as the chrM reference used in the GRCh38 human genome build [27]. The target depth of mtDNA sequencing was about 30,000× (mean = 38,847), which provided us with the ability to analyze these ultra-deep sequencing data for low-level mitochondrial heteroplasmy (Figure 1A).

### 2.2. Cutoff Determination and Variants Call

We compared the relationship between background error and VAF (variant allele frequency) distributions through simulation by using an in silico admixture of mtDNA-seq data from the HEK293 cell line and one clinical kidney tissue sample with CKD stage G1. We used HEK293 because it is derived from human embryonic kidney [28]. There were 13 homoplasmic variants shared between HEK293 and the kidney sample, while 23 homoplasmic variants were specific to the kidney sample (Figure 1B). HEK293 and the kidney sample data were mixed at 2%, 1%, 0.5% and 0.25% ratios to artificially reproduce heteroplasmy at the 23 private positions. The coverage tracks of HEK293 and simulated admixture samples were calculated, and non-reference variants were defined by the percentage of reads without a reference base (see Section 4). Non-reference variants detected in HEK293 DNA at all mtDNA positions should mainly reflect background errors as well as include homoplasmic and heteroplasmic variants present in the cell line. Therefore, we calculated the upper limit of background error (0.00204) based on the nonparametric outlier determination method (see Section 4). On the other hand, the simulated admixture follows a normal distribution centered at a particular mixture ratio. Hence, the lower confidence limit for each mixture ratio (2% = 0.01474, 1% = 0.00757, 0.5% = 0.00376, 0.25% = 0.00178) was calculated as the lower threshold of the distributions at the artificial heteroplasmic positions using a parametric method (see Section 4). The lower confidence limit of the 0.25% mixture crossed the upper fence determined for the background error, while the 0.5% mixture did not, suggesting that analyses using 0.25% as a VAF cutoff would contain more false-positive variants. Hence, we defined 0.5% as the minimum VAF cutoff for calling mtDNA variants (Figure 1C). As a result, a total of 697 SNVs and 504 indels were detected as being heteroplasmic (0.5% ≤ VAF < 95%) (Tables S1 and S2).

### 2.3. Accumulation of Heteroplasmic SNVs and Indels

**Figure 2:**
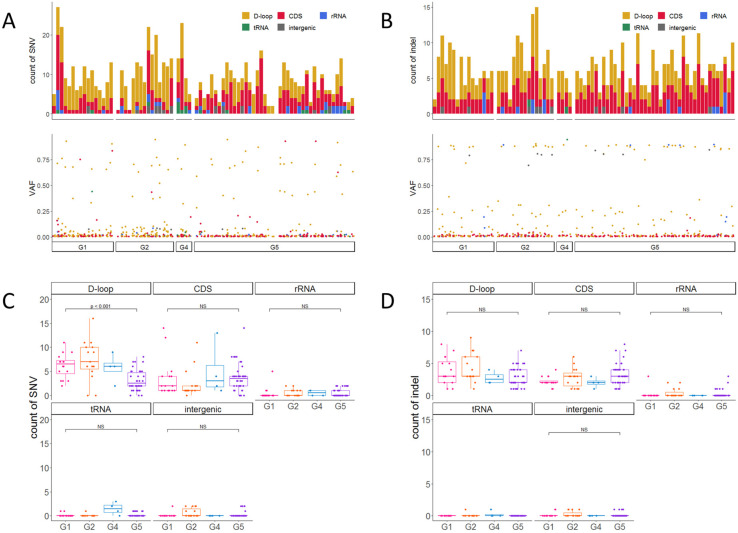
Count of detected variants (top panel) and VAF distribution (bottom panel) of SNVs (**A**) and indels (**B**) per sample. Bars and dots are colored by region annotation. Counts of region-specific SNV (**C**) and indel (**D**) variants. Colors were differed by CKD stages. Significance was evaluated using Dunnett’s test.

To investigate mtDNA damage that might accumulate with CKD progression, we first compared the number of SNV and indel variants with VAF lower than 95% between CKD stages, but there was no significant difference in the variant number among CKD stages (*p* = 0.2 and *p* = 1.0, G1 vs. G5, using Dunnett’s test) (Figure S1A,B). Next, we examined whether variants in different mtDNA region types were differentially mutated with CKD progression (Figure 2A,B top) and found significant decreases in the number of SNVs in the D-loop region (*p* < 0.001, using Dunnett’s test) when comparing CKD stages G1 to G5. Similarly, there were statistically significant trends for decreased SNV number in the D-loop (*p* < 0.001, using the Jonckheere–Terpstra test) and increased indels in CDS (*p* < 0.05, using the Jonckheere–Terpstra test) with CKD progression (Figure 2C,D). All of the indels in CDS were frameshift variants. To examine heteroplasmy levels, especially low-level heteroplasmy, we then evaluated the VAF of SNVs and indels (Figure 2A,B bottom).

Most variants exhibited low-level heteroplasmy (VAF 90th percentile = 0.294) (Figure S2A), so we then focused on the variants with a frequency of less than the 90th percentile to examine whether low-level heteroplasmic variants differed according to CKD stage. However, the VAF distribution did not differ between stages G1 and G5 (*p* = 0.35, using the Kruskal–Wallis test) (Figure S2B). Somatic variants are believed to accumulate with age [29]. In this mtDNA study, no correlation was found between age and SNVs (*p* = 0.1) or indels (*p* = 0.9) (Figure S3). Together, these results suggest that variants do not accumulate at a mtDNA genome-wide level with CKD progression, but these variants may differentially accumulate depending on the mtDNA region type.

### 2.4. Mutational Signatures of mtDNA

We analyzed the accumulated mtDNA variants in regard to their mutational signatures and found that most SNVs were transition mutations (C > T or T > C) (Figure S4A), which is consistent with the previous result of the pan-cancer whole-genome data [8]. We then compared the signatures in mtDNA against COSMIC signatures using sigminer [30]. The single base substitution (SBS) signature with the highest similarity was SBS5, which is associated with clock-like mutational mechanisms, but the similarity score was low (cosine similarity = 0.550). SBS18 and SBS36, which are related to reactive oxygen species (ROS) signatures, were not detected (Figure S4B) [31,32]. Indel (ID) signatures possessed somewhat high similarity to the COSMIC ID2 signature (cosine similarity = 0.792), which has a proposed etiology of slippage during DNA replication of the template DNA strand (Figure S4C). However, this similarity was detected across all CKD stages (Figure S4D).

### 2.5. Homoplasmic mtDNA Variants

**Figure 3:**
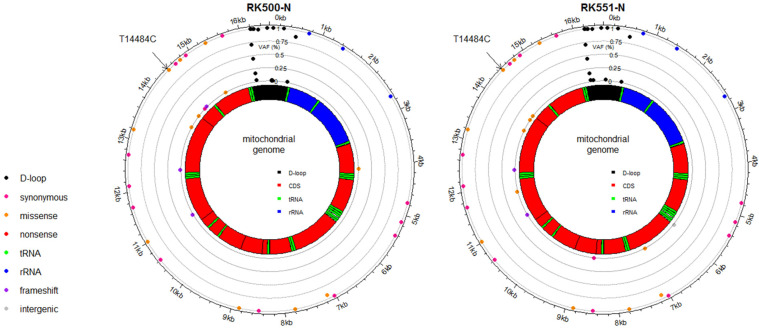
Distribution of mtDNA variants in RK500-N (**left panel**) and RK551-N (**right panel**) with T14484C homoplasmy. Outer scale represents heteroplasmy level. Each color dot corresponds to the type of variant.

To assess the risk of mitochondrial homoplasmy for CKD, we focused on the variants with 95% or greater VAF. We compared CKD G2-G5 with G1 patients and observed a statistically significant decrease in the T16304C alleles in CKD patients (*p* < 0.01, using a chi-squared test) (Figure S5). In addition, one patient with CKD stage G5, from whom kidney tissues were obtained from both left and right kidneys due to the occurrence of two primary tumors (7 years apart), harbored the T14484C variant, which is associated with LHON (Leber’s hereditary optic neuropathy) and affects the structural components of NADH: ubiquinone oxidoreductase (Complex I) (Figure 3) [33]. That patient did not manifest the phenotype of LHON or other mitochondrial diseases, which may be explained by a previously reported connection between the penetrance of T14484C and mtDNA haplogroups [34]. 

### 2.6. Deleterious mtDNA Variants

**Figure 4:**
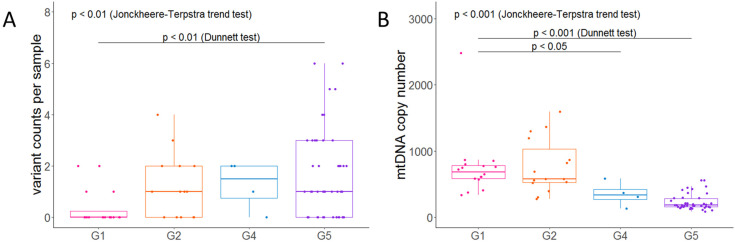
(**A**) Counts of variants annotated as deleterious variants. Deleterious variants include nonsense, frameshift, tRNA and confirmed pathogenic missense variants. Colors were differed by CKD stages. Significance was evaluated using Dunnett’s test, and any increasing trend was evaluated using Jonckheere–Terpstra test. (**B**) Comparison of mtDNA copy number in kidney tissue samples. The mtDNA copy numbers were evaluated using qPCR. Colors were differed by CKD stages. Significance was evaluated using Dunnett’s test and any decreasing trend was evaluated using Jonckheere–Terpstra test.

We annotated mtDNA variants using MITOMASTER [35] and selected missense variants that were confirmed as pathogenic via MitoPhen [36], as well as nonsense and frameshift variants as deleterious variants. Variants in tRNA genes were annotated using the MitoTIP score, and 5/15 possibly pathogenic variants were also included as deleterious variants [37]. Only the single variant, A3565AC, was significantly associated with advanced CKD (G2-G5; *n* = 21/61, G1; *n* = 0/16; *p* < 0.05, using a chi-squared test). However, the heteroplasmy levels for that variant were low (0.5–2.3%), and several variants were in homopolymer regions, which are vulnerable to DNA polymerase error in vitro and in vivo. We excluded odd indels, which were detected frequently, at the positions of 11,031 (59/77) and 12,417 (77/77) at the A7 or A8 homopolymers, regardless of CKD stage. Interestingly, the numbers of deleterious mtDNA variants per patient were higher in CKD G5 than G1 patients (*p* < 0.01, using Dunnett’s test) and increased with CKD progression (*p* < 0.01, using the Jonckheere–Terpstra test) (Figure 4A). Most deleterious variants were annotated as frameshifts, which showed a significant difference (*p* < 0.01, G1 vs. G5, using Dunnett’s test). Pathogenic missense, nonsense and tRNA variants were more frequent in patients with CKD G4 and G5, although the difference was not significant (Figure S6A,B). Most deleterious variants exhibited low-level heteroplasmy (0.5 ≤ VAF ≤ 1% = 55/73, G5) (Figure S7). In addition, the accumulation of deleterious variants was not significantly correlated with age (*p* = 0.3) (Figure S6C).

To evaluate the influences of cancer and the specificity of variants in kidney tissues, we additionally sequenced the mtDNA of 12 kidney cancer and 4 blood samples whose corresponding non-tumor kidney tissues were found to have at least two deleterious variants with low VAF. Among the 42 deleterious mtDNA variants, only 1 variant A11866AC in RK506 was increased in the tumor tissue (1.27% in non-tumor kidney, 21.37% in tumor, Tables S3 and S4). No variant was detected at higher than 0.5% VAF in the blood (Table S3). These results suggested that the deleterious mtDNA variants found in the CKD tissues were independent of variants in the tumors and also not inherited by the tumor cells.

### 2.7. Decreased mtDNA-CN in Kidney Tissue with CKD Progression

The abnormality of mtDNA-CN (copy number) is also involved in mitochondrial dysfunction and suggested to be a biomarker and risk factor for kidney diseases [38,39]. In CKD patients, decreased mtDNA-CN was reported in the blood and muscle [40,41,42]. However, mtDNA-CN in kidney tissue with CKD is not well-known. To evaluate mtDNA-CN, we performed quantitative PCR (qPCR) for ND1 and the nuclear gene, PPIA, and observed that mtDNA-CN significantly decreased with CKD progression (*p* < 0.001, G1 vs. G5, using Dunnett’s test, and *p* < 0.001, using the Jonckheere–Terpstra test) (Figure 4B). Then, we normalized the detected total SNVs and indels via mtDNA-CN and found that there were significance differences (*p* < 0.01 and *p* < 0.001, G1 vs. G5, using Dunnett’s test) and an increasing trend (*p* < 0.001, using the Jonckheere–Terpstra test) (Figure S8).

## 3. Discussion

Various mtDNA enrichment methods, such as REPLI-g, Agilent SureSelect and LRPCR, exist for mtDNA sequencing [43,44,45,46]. Of these methods, LRPCR has the advantage that it can be used to detect both point variants and small deletions, can detect mtDNA variants at low VAF [47], and has the added benefit that it can be customized for various levels of DNA quality [26]. To evaluate this high-fidelity method’s capacity to analyze mtDNA variants, even at low VAF, we performed LRPCR-based mitochondrial enrichment with three pairs of primers. This approach provided ultra-deep sequence data (30,000×) and enabled us to analyze low-level heteroplasmy. Nevertheless, it remains challenging to detect low-level heteroplasmic variants reliably [48]. Therefore, we simulated artificial heteroplasmy by mixing the sequence data from the monoclonal HEK293 cell line and one clinical sample and then determined an appropriate minimum VAF cutoff, considering the background error of HEK293 and the distribution of VAF values from simulated heteroplasmy. SNVs and indels were, respectively, called using mutserve and VarScan2 because mutserve provides the best performance to detect low-level heteroplasmic SNVs [49], but any standardized calling method for mtDNA indels has not yet been established. Indels in homopolymer regions have great potential for errors (10 or more) and being longer, and A/T repeats have more potential for errors, which result from slippage error in DNA polymerases [50]. However, we applied a unified analytic workflow for all samples and excluded only the indels detected commonly among the 77 samples, which were likely to result from these homopolymer errors.

Mitochondria generate the majority of the endogenously reactive oxygen species (ROS) in a cell, which can damage DNA and lead to a higher mutation rate in mtDNA than nuclear DNA. In addition, it has been suggested that environmental chemical exposure may contribute to accelerating mtDNA damage and the aging process. Hence, many types of mtDNA alterations, including copy number changes, have been identified in primary human cancers, the occurrence of which tends to increase with age [29,51]. In this study, we observed that deleterious low-level heteroplasmic mtDNA variants accumulated with CKD progression, whereas we did not obtain any evidence that the overall mutation rate of mtDNA increased. But COSMIC SNV signatures, SBS18 and SBS36, which are associated with damage by ROS, were not detected [31,32]. This result may be attributed to these signatures being based on the whole-genome sequencing (WGS) and whole-exome sequencing (WES) of nuclear genomes. In consideration of this, different analytical systems, divorced from those derived from nuclear DNA analyses, may be necessary when we analyze mtDNA for mutational signatures [52,53]. Taking account of the genome length, mtDNA may be too small for signature analysis. On the other hand, the mutational spectrum resembled that previously seen for mtDNA in kidney cancer [8].

The kidney requires a large number of mitochondria to function [14]. MtDNA-CN is maintained by the synthesis and degradation rates of mtDNA [54]. Changes in mtDNA-CN directly correlate with energy production and precede the development of various chronic diseases [55]. In this study, we observed that mtDNA-CN decreased in kidney tissues with advancing CKD stage. This result is similar to previous studies that showed a decreased mtDNA content in the blood and muscle in patients with CKD [40,41,42]. A study of the renal cortex of a rat partial nephrectomy model of chronic renal failure also supports our result in human kidney tissue and suggested that mitochondrial biogenesis was not stimulated [56]. Mitophagy, which is one of the mitochondrial quality control systems, might be more dominant than mitochondrial biogenesis [14,54,57].

More deleterious variants were detected in advanced CKD patients. Nevertheless, low-level heteroplasmic variants may have little physiological impact. Although different functional units of the kidney show different aerobic and anaerobic energy generation profiles depending on the region or cell types [58,59], bulk sequencing data cannot determine which cells carry variants. Our results, showing no differences in heteroplasmy shifts and counts of total SNV or indel variants, might be supporting evidence of correctly functioning mitochondrial quality control mechanisms [10]. However, mitophagy might selectively degrade dysfunctional mitochondria to prevent the accumulation of harmful mitochondria, thereby leading to mtDNA-CN decrease [60]. Although mtDNA variants affecting mitochondrial functions and copy number changes may be associated with CKD progression, it is unclear what mechanisms are engaged in kidney dysfunction.

There are several limitations in this study. First is the detection specificity for low-VAF variants, especially indels, and it is still difficult to discriminate actual low-VAF variants from background errors. Second is cellular heterogeneity. Kidney tissue consists of many types of cells, and we do not know which cells contain mtDNA variants and cause mitochondrial dysfunction in CKD tissues. Third is that it is unclear how these low-level heteroplasmic variants can affect renal dysfunction, or if they are just the result of CKD progression. Fourth is that we analyzed kidney tissue around the kidney cancer, and cancer may be able to affect kidney function and mtDNA variants in non-tumor kidney tissues.

## 4. Materials and Methods

### 4.1. Clinical Samples

Seventy-seven frozen non-tumor kidney tissues were obtained from seventy-six patients (two samples came from one individual) who underwent total nephrectomy for kidney cancer at Iwate Medical University Hospital and Yokohama City University Hospital. We harvested normal kidney tissues that were located farthest from the tumors in resected kidneys (total nephrectomy). In partial nephrectomy, pathologists check, microscopically, that the margins are free of the resected tumor, and we harvested normal kidney tissue farthest from the tumor in resected specimens, which did not contain the tumor tissue, macroscopically. This study was approved by the Institutional Review Board of Iwate Medical University Hospital, Yokohama City University, and RIKEN, and each patient provided written informed consent for the publication of this report. Each patient was assessed for their renal function, eGFR (estimated Glomerular Filtration Rate), before surgical resection, and their stage of CKD was classified (G1: *n* = 16, G2: *n* = 15, G4: *n* = 4, G5: *n* = 42) according to the KDIGO CKD guideline [61]. Twenty-seven patients with end-stage renal disease or receiving dialysis were included [20]. Patient information is listed in Table S5.

### 4.2. HEK293 Cell Culture

The HEK293 cell line was obtained from ATCC (CRL-1573™) and cultured from frozen stocks at 37 °C in Minimum Essential Media (MEM: Gibco) supplemented with 10% (*v*/*v*) heat-inactivated fetal calf serum (FBS: Gibco), 100 U mL^−1^ penicillin, 100 ug mL^−1^ streptomycin and 250 ng mL^−1^ amphotericin B (Sigma-Aldrich, St. Louis, MO, USA).

### 4.3. DNA Extraction

Total DNA was extracted using the QIAamp DNA Mini Kit (QIAGEN, Hilden, Ger-many), and the concentration was measured via fluorometric quantification using the In-finite F Nano+ (TECAN, Mannedorf, Switzerland) and the Quant-iT™ PicoGreen^®^ dsDNA Assay Kit (Thermo Fisher Scientific, Waltham, MA, USA).

### 4.4. PCR-Based mtDNA Enrichment and Sequencing

The mtDNA was amplified by three pairs of primers listed in Table S6 that were reported in a previous study [26]. PCR was performed using KOD One^®^ PCR Master Mix (Toyobo, Osaka, Japan). A total of 50 ng of extracted DNA was used as a template in a 50 μL PCR volume. The PCR conditions included 98 °C incubation for 2 min followed by 30 cycles of a 10 s denaturation step at 98 °C and a 35 s annealing and extension step at 68 °C. There was one final extension step at 68 °C for 10 min followed by infinite holding at 4 °C. Electrophoresis was performed using 1 μL of PCR products and 1% agarose gel (NIPPON GENE, Tokyo, Japan) to check for specific amplification products. PCR products were purified using MultiScreen (Merck KGaA, Darmstadt, Germany), and the concentrations were measured using the Agilent 2100 Bioanalyzer and the High Sensitivity DNA Kit (Agilent, Santa Clara, CA, USA). Then, all amplicons from the same template DNA were equimolarly pooled, and 200 ng of pooled DNA was fragmented into about 200 bp fragments using a Covaris ultrasonicator. mtDNA libraries were constructed using a KAPA Hyper Prep kit (KAPA Biosystems, Wilmington, MA, USA), and the concentrations of completed libraries were measured using Agilent 2200 TapeStation and D1000 ScreenTape (Agilent). Finally, 125 bp paired-end sequencing of the mtDNA libraries was performed using Illumina HiSeq2500 SBS version 4 (Illumina, San Diego, CA, USA).

### 4.5. Variant Calling

Fastq files were trimmed with Cutadapt (v3.4) and aligned with the revised Cambridge Reference Sequence (rCRS, GenBank: NC_012920.1) with BWA-MEM (v0.7.17) [27,62,63]. SAMtools (v1.9) was used to create BAM files, and PCR duplicates were removed with Picard’s MarkDuplicates function (v2.25.0) (http://broadinstitute.github.io/picard/, accessed on 5 December 2023) [64]. The depth of mtDNA sequencing was calculated using samtools (v1.13) [64]. SNVs were called using mutserve (v2.0.0-rc13: --level 0.001, --baseQ 30) and indels were called using VarScan2 (v2.3.9: mpileup2indel –min-var-freq 0.001 –min-avg-qual 30 –output-vcf) [65,66]. We filtered out false-positive variants due to misalignment to poly-C stretch (302–315) and 3107N (ACNTT, 3105–3109) in all samples. Homoplasmic deletions were found in 14 samples (248 del) and 1 sample (16,256 del). By visually inspecting variants using IGV [67], we found that these deletions resulted in the false-positive detection of SNVs at these positions, so we excluded SNVs at positions 248 and 16,256 in individuals with homoplasmic deletions.

### 4.6. In Silico Admixture

We identified homoplasmic variants (VAF ≥ 95%) of HEK293 and the clinical sample (BHD-F123-N) and used 23 variants that were private to BHD-F123-N to simulate artificial heteroplasmic sites through an in silico admixture of HEK293 and BHD-F123-N mtDNA-seq data. BAM files were split at file size ratios of 98:2, 99:1, 99.5:0.5 and 99.75:0.25 (HEK293:BHD-F123-N) and merged using SAMtools (view -s and merge). The coverage tracks of HEK293 and admixtures were counted with igvtools (v2.10.2 count -w 1 –bases), and the VAF of non-reference variants was calculated using the following equation:

VAF of non-reference variants = (Total read ct. − Read ct. of reference base)/Total read ct.,(1)

The upper fence (limit) for VAF due to sequence analysis errors was determined from nonparametric outlier analysis of the distribution of VAF across all mtDNA non-reference variants observed in HEK293 using the following equation:

Upper fence = 3rd quartile + 1.5 × interquartile range,(2)

The lower confidence limits of admixtures were determined by the VAF at 23 artificial heteroplasmic sites using the following equation:

Lower confidence limit = mean − 3 × standard deviation,(3)

### 4.7. Mutational Signature Analysis

Mutational spectrums were analyzed using R from vcf files by extracting and visualizing single base substitution (SBS) and indel (ID) mutational signatures using sigminer (v2.1.9) [30].

### 4.8. Variant Annotation

The mtDNA region information was loaded from the GenBank file using the genbankr and GenomicRanges (cds and otherFeatures function) R packages. The components of “start”, “end” and “type” were extracted; then, “misc_feature” was filtered out and D-loop (1–576, 16,024–16,569) was manually added. Finally, regions lacking features were listed as “intergenic”. The “type” annotation was merged into the variants based on the variant positions between “start” and “end”. Furthermore, SNVs were annotated using MITOMASTER [35], and then, the output files and VCF files of SNVs and indels were processed using R. Missense and nonsense variants were classified based on the amino acid substitution listed in the output files of MITOMASTER. Frameshift variants were classified as those located in CDS for which the number of inserted/deleted bases was not divisible by three. Missense variants were considered deleterious if they were listed as “confirmed” pathogenic variants via MitoPhen [36]. tRNA pathogenicity was evaluated using the MitoTip score [37], and tRNA was interpretated as “confirmed pathogenic”, “likely pathogenic” and “possibly pathogenic” and included as deleterious variants. Indels detected at the positions of 11,031 and 12,417 were excluded due to suspected errors in the A7 or A8 homopolymer. Detected variants were checked as to whether they were reported in ChRCC [68]. All SNVs and indels detected by this analysis are listed in Table S1 and S2.

### 4.9. Evaluation Using Cancer and Blood

The mtDNA libraries of kidney cancer and blood were constructed using the same methods and sequenced using Illumina NextSeq 2000 P1 (Illumina). Vcf files were created using the same tools with a 0.1% cutoff. The evaluation of the VAF data of kidney cancers is listed in the Table S3. The mtDNA variants with VAF > 10% are also listed in the Table S4.

### 4.10. mtDNA Copy Number (mtDNA-CN)

The mtDNA and nuclear DNA were analyzed using an Applied Biosystems^®^ 7900HT (Applied Biosystems, Thermo Fisher Scientific). PCR was performed using the KAPA SYBR^®^ FAST qPCR Kits (KAPA Biosystems). The primers and DNA template were added according to the instructions, and the following program was used: 1 × 2 min at 50 °C; 1 × 10 min at 95 °C; 40 × 15 s at 95 °C for denaturation; 1 min at 65 °C for annealing and extension. For mtDNA, primers for the ND1 gene were used, and for nuclear DNA, primers for the PPIA gene were used (Table S7). mtDNA-CN was calculated using the following equation:

mtDNA copy number (mtDNA-CN) = 2 ^ (*PPIA* Ct value − *ND1* Ct value), (4)

### 4.11. Statistical Analysis

Statistical tests were conducted within R and using the clinfun and multcomp packages. Group-to-group differences were assessed using Dunnett’s test or the Kruskal–Wallis test for parametric or nonparametric continuous values and a chi-squared test for discrete values. Any trend was evaluated using the Jonckheere–Terpstra test with respective alternatives. Significance levels for each test are indicated in the figures.

## Data Availability

Raw sequence data presented in this study are available in NBDC under the accession number JGAS000611.

## Supplementary Materials

The following supporting information can be downloaded at: https://www.mdpi.com/article/10.3390/ijms242417212/s1.
