# Supplementary material for: Mitochondrial DNA Variants at Low-Level Heteroplasmy and Decreased Copy Numbers in Chronic Kidney Disease (CKD) Tissues with Kidney Cancer
Source: Int J Mol Sci. 2023 Dec 7;24(24):17212. doi: 10.3390/ijms242417212 (PMC10743237; doi:10.3390/ijms242417212)
Supplement: Supplementary file 1 [file ijms-24-17212-s001.zip › ijms-2635534-supplementary/Supplementary Materials Figures S1-S8.pdf]

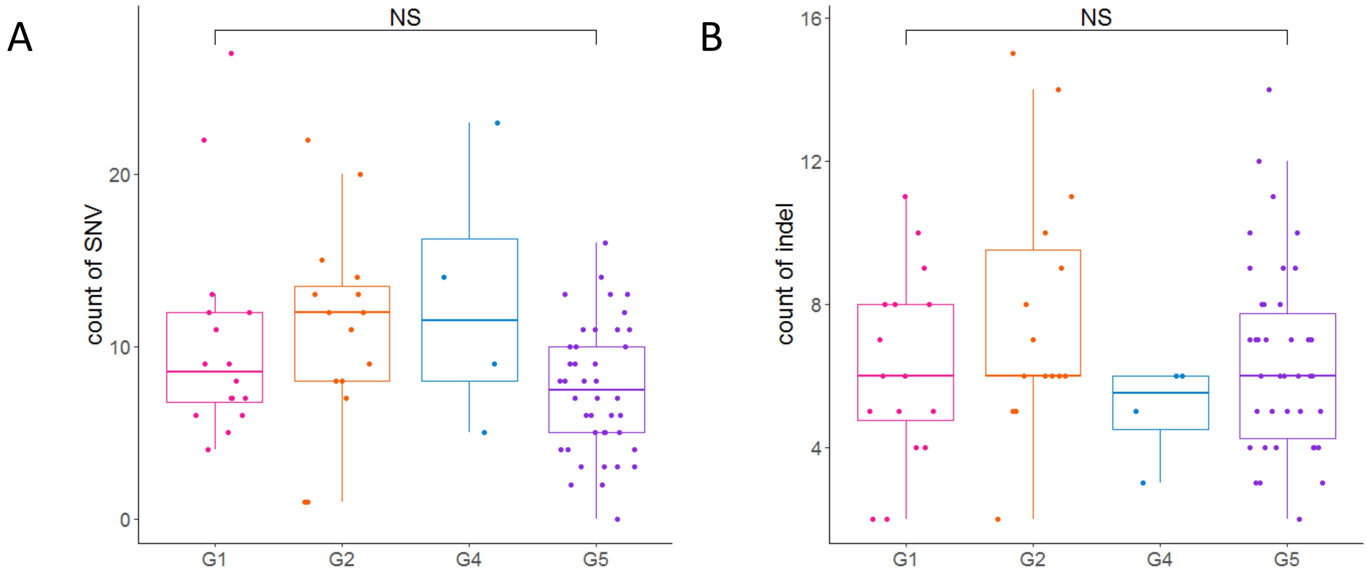

Supplementary Figure S1. Comparison of called variants among the CKD stage. Counts of all called SNV (**A**) and indel (**B**) variants. Colors were differed by CKD stages. Significance was evaluated with Dunnett's test.

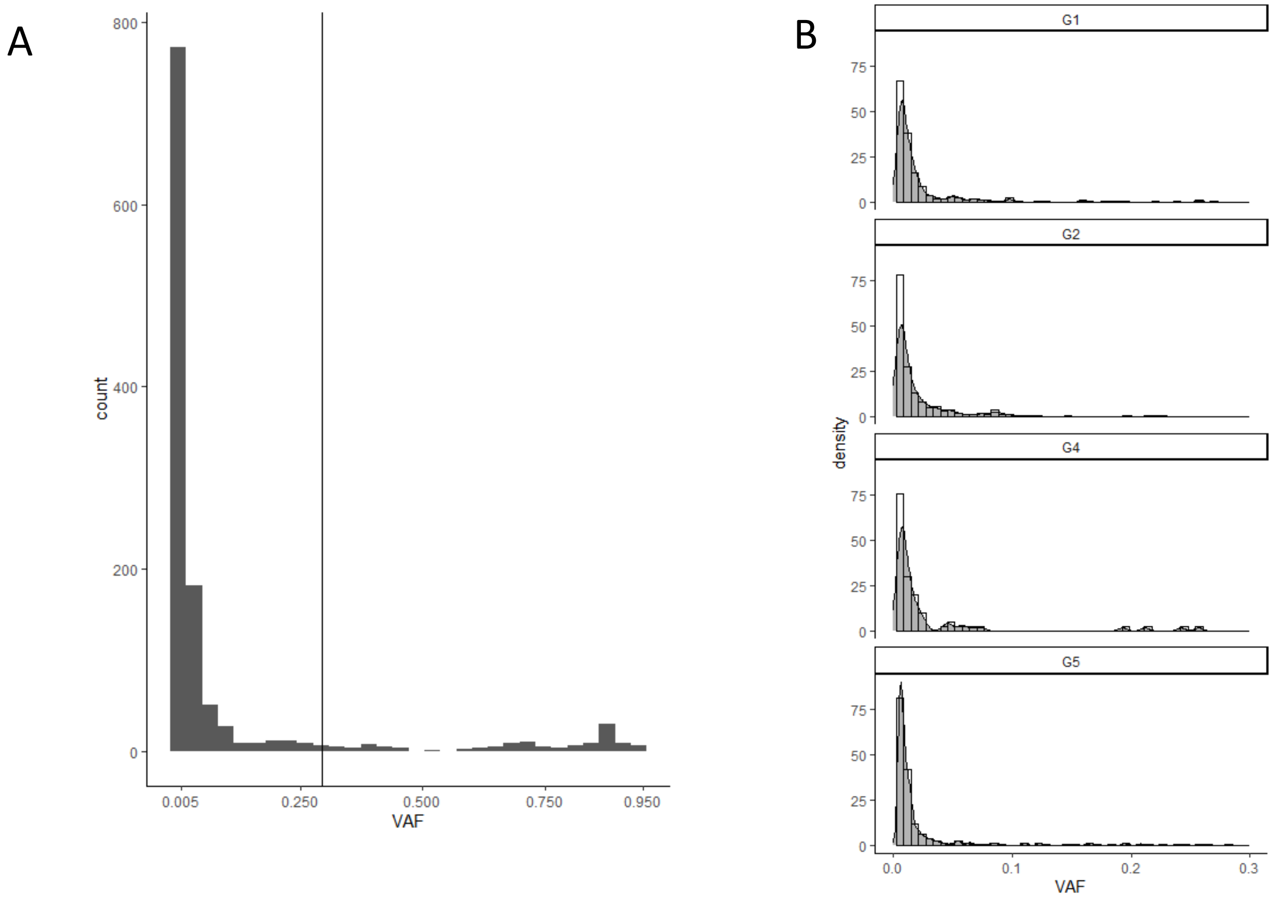

Supplementary Figure S2. VAF distribution of SNVs and indels. (**A**) VAF distribution of all samples. Vertical line indicates the 90th percentile (0.129). (**B**) VAF distribution under 90th percentile cutoff (0.129) per CKD stage.

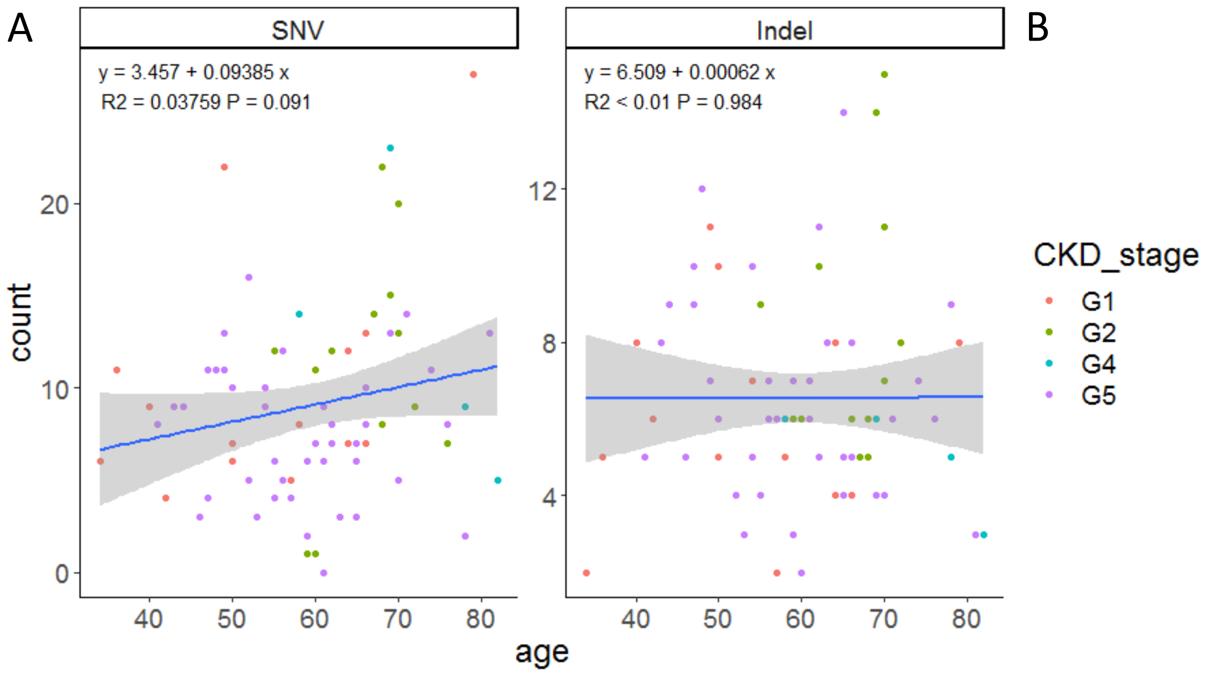

Supplementary Figure S3. Correlation between age and variant counts Linear regression provided the relationship between the age and SNVs (**A**) or Indels (**B**). Colors were differed by CKD stages.

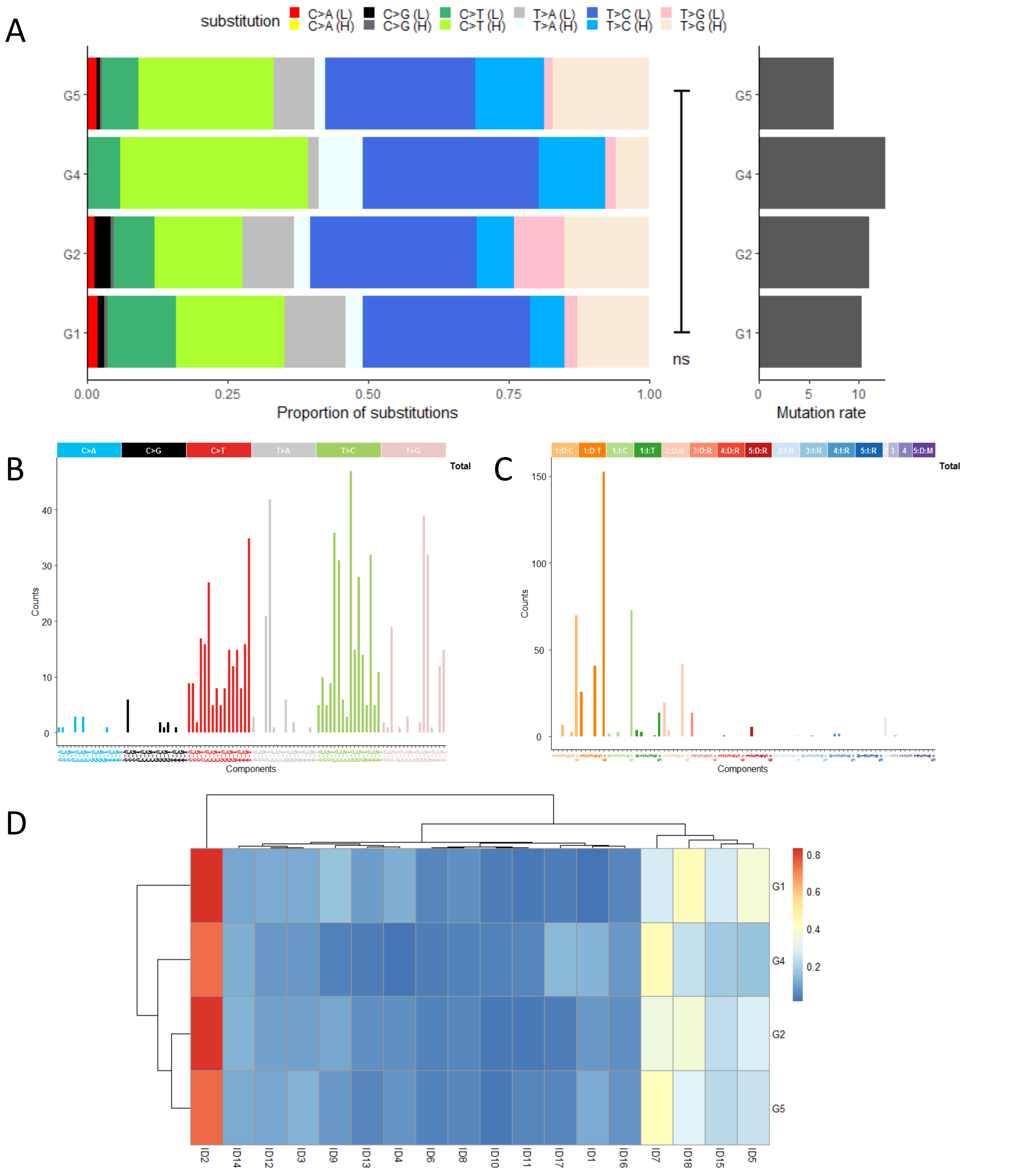

Supplementary Figure S4. Mutation landscapes. (**A**) Mutation spectrum of mtDNA from CKD. The P values were calculated by chi-squared test without multiple comparison adjustment. Mutation rates were generated by the mean number of substitutions per sample. (**B**) SBS signature extracted from all clinical samples. (**C**) ID signature extracted from all clinical samples. (**D**) ID signatures across the CKD stage.

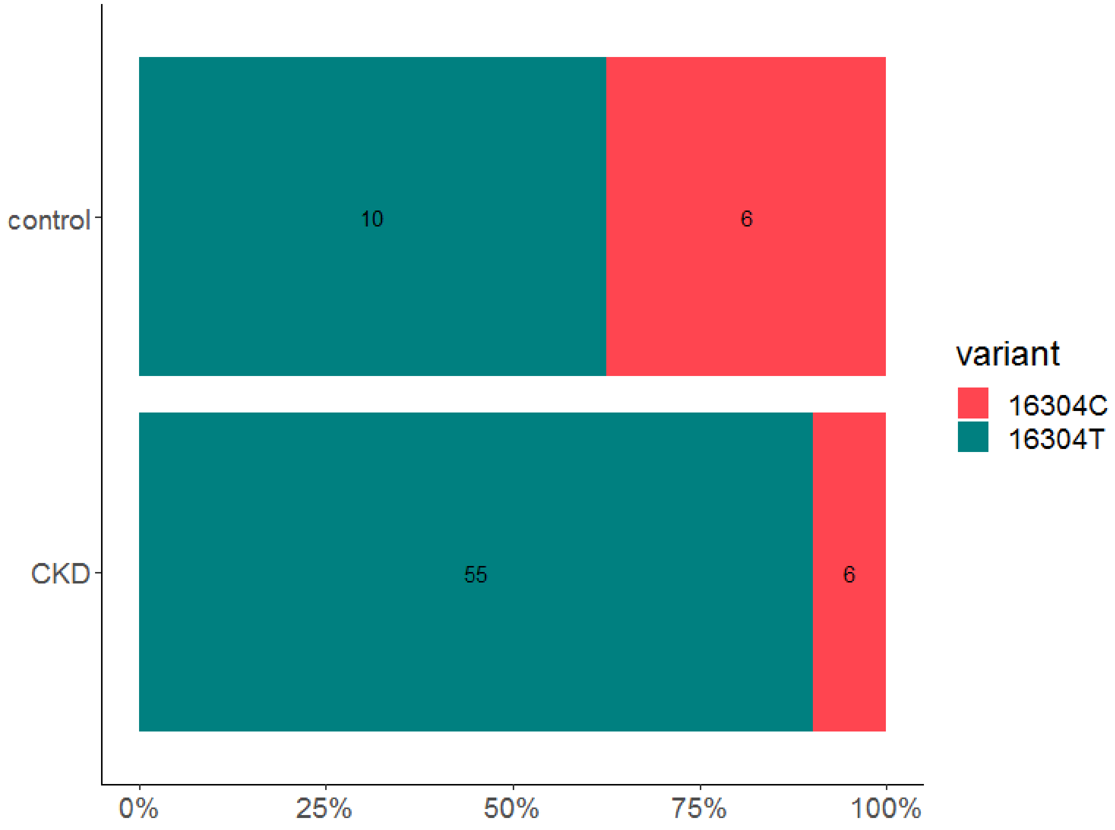

Supplementary Figure S5. Frequency of homoplasmy in 16304.
Stacked bar plots of the frequencies of the homoplasmic variant at 16304 in control and CKD. The control means the patients with CKD stage G1 and CKD includes the patients with CKD stage G2, G4 and G5.

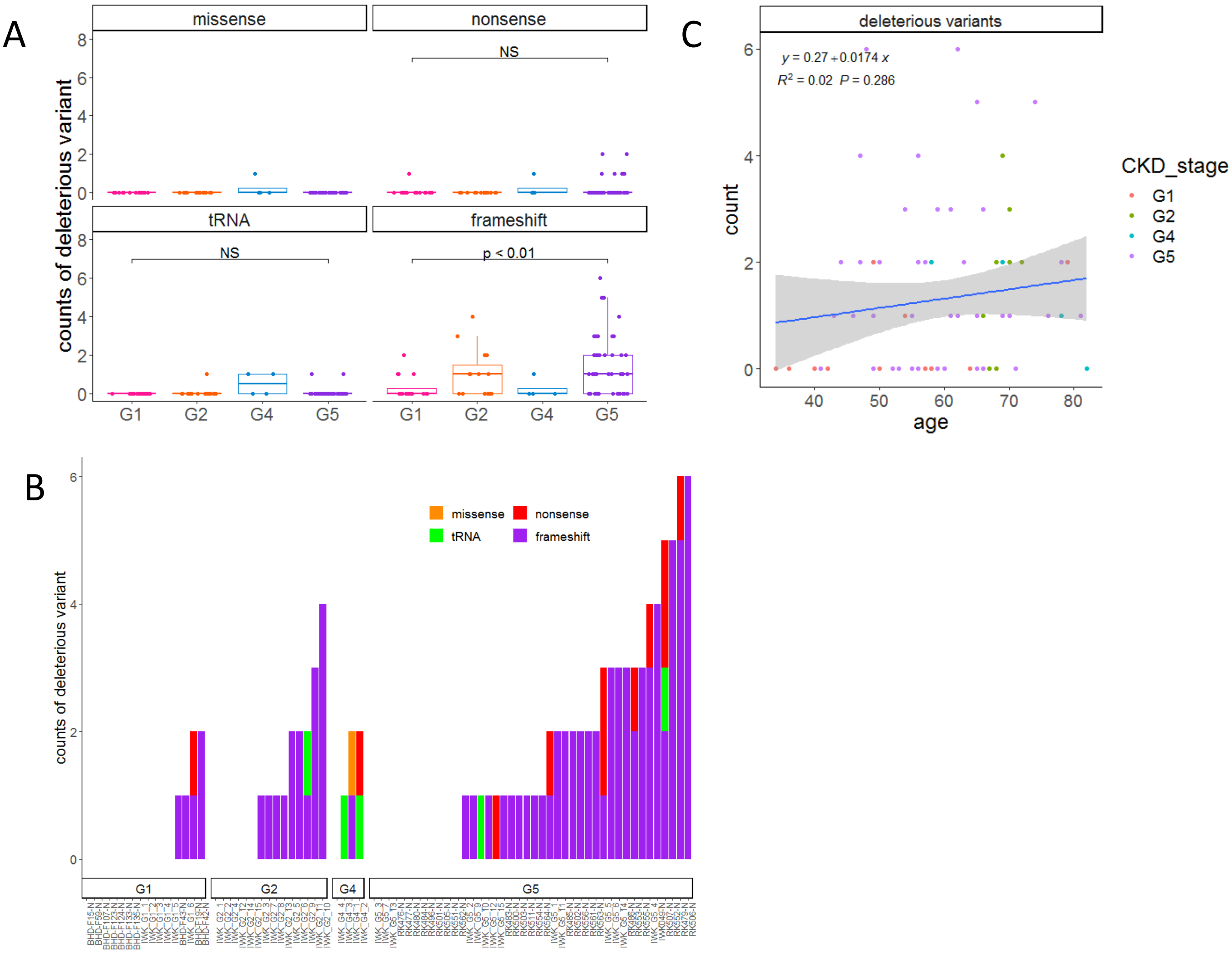

Supplementary Figure S6. Additional profiles of deleterious variants. (**A**) Counts of variant per the kind of deleterious annotations. Colors were differed by CKD stages. Significance was evaluated with Dunnett's test. (**B**) Counts of deleterious variants per sample. Each color represents the kind of deleterious annotations. (**C**) Correlation between age and the counts of deleterious variants. Colors were differed by CKD stages.

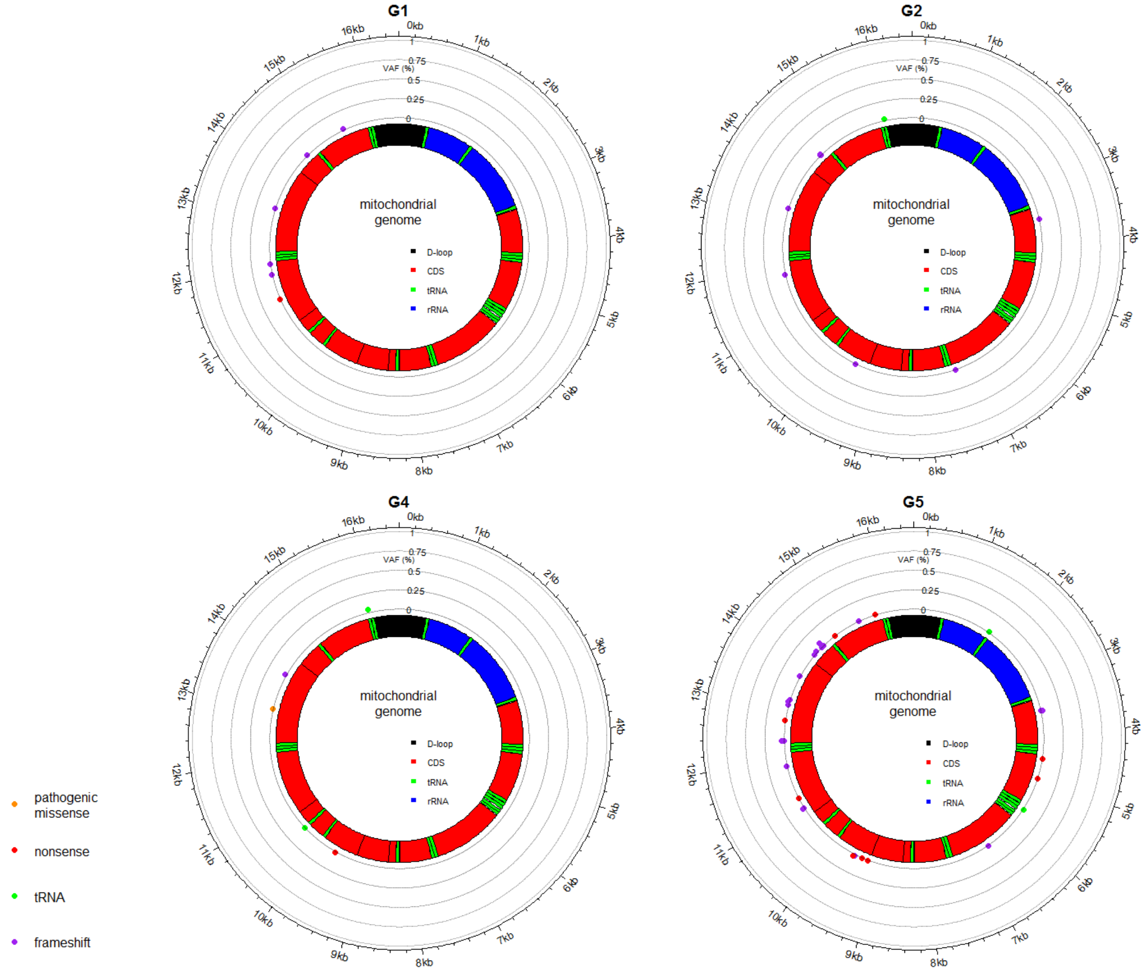

Supplementary Figure S7. VAF of the deleterious variants across the CKD stage. Outer scale represents heteroplasmy level. Each color dot corresponds to a type of variants.

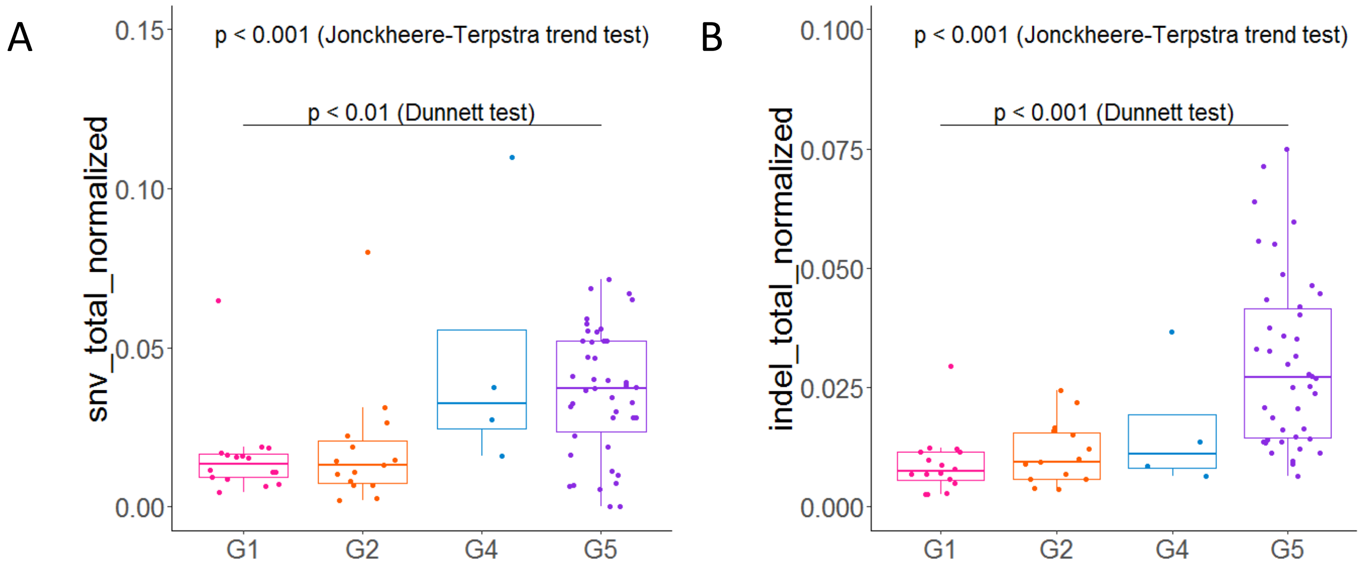

Supplementary Figure S8. Counts of all called SNV (**A**) and indel (**B**) variants normalized by mtDNA copy number. Colors were differed by CKD stages. Significance was evaluated with Dunnett's test and increasing trend was evaluated with Jonckheere-Terpstra test.
